# Miscellany

**Published:** 1892-04

**Authors:** 


### Miscellany.

---

### THE PAN-AMERICAN MEDICAL CONGRESS.

*Circular of Information.* [*Official.*]

THE Committee on Permanent Organization met at St. Louis, October 14, 15, and 16, 1891, and adopted a series of General Regulations for the permanent organization of the Pan-American Medical Congress, and a series of Special Regulations for the government of the first meeting, and recommended that the incorporators adopt both series of regulations as the organic law of the Congress.

Pursuant to such regulations, the following general officers were elected, viz.: William Pepper, M. D., LL. D., Philadelphia, Pa., *President;* Abraham M. Owen, A. M., M. D., Evansville, Ind., *Treasurer;* Charles A. L. Reed, M. D., Cincinnati, Ohio, *Secretary-General.*

INTERNATIONAL EXECUTIVE COMMITTEE—*Argentine*, Dr. Pedro Lagleyze; *Bolivia*, Emilio de Tomassi; *Brazil*, Dr. Carlos Costa; *British North America*, Dr. James F. W. Ross; *British West Indies*, Dr. James A. De Wolf; *Chili*, Dr. Moises Amaral; *Colombia*, P. M. Ibañez; *Costa Rica*, Dr. D. Nuñez; *Ecuador*, Dr. Ricardo Cucalon; *Guatemala*, Dr. José Monteris; *Haiti*, Dr. D.

Lamothe ; *Hawaii*, ————; *Spanish Honduras*, Dr. George Bernhardt ; *Mexico*, Dr. Thomas Noriéga ; *Nicaragua*, Dr. Juan I. Urtecho ; *Paraguay*, ———— ; *Peru*, Dr. José Cassamira Ulloa ; *Salvador*, Dr. David J. Guzman ; *Santo Domingo*, ———; *Spanish West Indies*, Dr. Juan Santos Fernandez ; *United States*, Dr. A. Vander Veer ; *Uruguay*, Dr. Jacinto De Leon ; *Venezuela*, Dr. Elias Roderiguez ; *Danish, Dutch, and French West Indies*, —.

The Auxiliary Committee, nominated by the various members of the Committee on Permanent Organization each for his own State, and already commissioned by the Chairman, was confirmed.

The election of officers of sections was begun, but time would not permit of the completion of the list, which was referred to a special committee with power to act. It has been deemed inexpedient to publish the list until it is completed, which can hardly be accomplished before the meeting of the Committee on Permanent Organization, at Detroit, in June ; but the organization of particular sections will be announced through the medical press as rapidly as officers are elected by the special committee.

In accordance with the wish of the Committee on Permanent Organization, as expressed in Special Regulation No. 4, Drs. I. N. Love, A. B. Richardson, L. S. McMurtry, R. B. Hall, T. V. Fitzpatrick, and Charles A. L. Reed met in Cincinnati and signed the legal form of application for articles of incorporation of the Pan-American Medical Congress, which Articles of Incorporation were duly issued by the Secretary of the State of Ohio, under date of March 15, A. D. 1892.

At a meeting of the Incorporators, held March 16, 1892, the following regulations, general and special, recommended by the Committee on Permanent Organization, were formally adopted as the organic law of the Pan-American Medical Congress in accordance with the Laws of Ohio, and all elections had by the Committee on Permanent Organization, in accordance with such regulations, were confirmed and made a part of the laws of the Congress :

### General Regulations.

### TITLE.

1. This organization shall be known as the Pan-American Medical Congress, and shall meet once in —— years.

### MEMBERSHIP.

2. Members of the Congress shall consist of such members of the medical profession of the Western Hemisphere, including the West Indies and Hawaii, as shall comply with the special regulations regarding registration, or who shall render service to the Congress in the capacity of Foreign Officers.

OFFICERS.

3. The Executive Officers of the Congress shall be residents of the country in which the Congress shall be held, and shall consist of one President, such Vice-Presidents as may be determined by special regulations, one Treasurer, one Secretary-General, and one Presiding Officer and necessary Secretaries for each section, all of whom shall be elected by the Committee on Organization, and there shall be such Foreign Vice-Presidents, Secretaries, and Auxiliary Committees as are hereinafter designated.

THE COMMITTEE ON ORGANIZATION.

4. The Committee on Organization shall be appointed by the representative medical association of the country in which the Congress shall meet. This Committee shall select all domestic officers of the Congress, and shall at its discretion confirm all nominations by members of the International Executive Committee, and in the event that any member of the International Executive Committee shall fail to nominate by the time specified by special regulation, the Committee on Organization shall elect officers for the country thus delinquent. It may appoint Vice-Presidents and Auxiliary Committeemen in foreign countries independently of nominations by the members of the International Executive Committee. It shall appoint Auxiliary Committees, arrange for the meeting, and frame special regulations for the session of Congress for which it was appointed. It shall make a report of its transactions to the opening session of the Congress.

THE INTERNATIONAL EXECUTIVE COMMITTEE.

5. There shall be an International Executive Committee, which shall be appointed by the first Committee on Organization, and which shall consist of one member for each constituent country. This Committee shall hold permanent tenure of office, except that when a member shall fail to be present at a meeting of the Congress, his office shall be declared vacant and the vacancy be filled by election held by the registered members from the country from which he was accredited. In the event of no representation whatever from the country in question, the members of the International Executive Committee present shall determine what disposition shall be made of the office.

It shall be the duty of each member of the International Executive Committee to nominate from the medical profession of his country, one Vice-President for the Congress and one Secretary for each Section of the Congress, and to forward the same to the Chairman of the Committee on Organization; except that in any country in which the Congress shall meet, it shall be the duty of the member of the International Executive Committee for that country to request his representative national medical association to appoint a Committee on Organization, which Committee on Organization shall discharge the duties designated in Regulation IV. Members of the International Executive Committee shall also nominate such Auxiliary Committees, and shall furnish such information as the Committee on Organization may request.

6. The Committee on Organization may at its discretion cause the Congress to be incorporated, which incorporation shall hold only until the final disbursement of funds for the session held in that particular country. In the event of such incorporation, such officers shall be elected and in such manner as may be required by law.

7. The following shall be considered as the constituent countries of the Pan-American Medical Congress :

Argentine Republic, Bolivia, Brazil, British North America, British West Indies (including B. Honduras), Chili, Honduras (Sp.), Mexico, Nicaragua, Paraguay, Peru, Salvador, Colombia, Costa Rica, Ecuador, Guatemala, Haiti, Hawaiian Islands, Santo Domingo, Spanish West Indies, United States, Uruguay, Venezuela, Danish, Dutch, and French West Indies.

8. The Sections of the Congress shall be as follows :

(1) General Medicine, (2) General Surgery, (3) Military Medicine and Surgery, (4) Obstetrics, (5) Gynecology and Abdominal Surgery, (6) Therapeutics, (7) Anatomy, (8) Physiology, (9) Diseases of Children, (10) Pathology, (11) Ophthalmology, (12) Laryngology and Rhinology, (13) Otology, (14) Dermatology and Syphilography, (15) General Hygiene and Demography, (16) Marine Hygiene and Quarantine, (17) Orthopedics, (18) Diseases of the Mind and Nervous System, (19) Oral and Dental Surgery, (20) Medical Pedagogics, (21) Medical Jurisprudence.

LANGUAGES.

9. The languages of the Congress shall be Spanish, French, Portuguese, and English.

AUXILIARY COMMITTEES.

10. The Auxiliary Committee shall consist of one member for each medical society or one for each considerable center of population in each of the constituent countries of the Congress. Nominations for the Foreign Auxiliary Committee shall be made to the Chairman of the Committee on Organization by the members of the International Executive Committee, each for his own country, except that in the country in which the Congress is to be held nominations shall be made by the Committee on Organization. Appointments on the Auxiliary Committee shall hold only for the meeting for which they were made.

Members of the Auxiliary Committee shall be the official representatives of the Congress in their respective localities. It shall also be their duty :

(1) To transmit to the profession of their respective districts all information relative to the Congress forwarded to them for that purpose by the general officers.

(2) To coöperate with the officers of sections in securing desirable contributions to the proceedings of the Congress.

(3) To furnish to the general officers such information as they may request for the purpose of promoting the interests of the Congress.

(4) To cause such publicity to be given of the development of the organization as will elicit the interest of the profession and secure attendance upon the meeting, and they shall discharge such other duties as will promote the welfare of the Congress.

### Special Regulations of the first Congress.

### TIME AND PLACE OF MEETING.

1. The first Pan-American Medical Congress shall be held in the City of Washington, D. C., September 5, 6, 7, and 8, A. D. 1893.

### REGISTRATION.

2. The Registration fee shall be $10.00 for members residing in the United States, but no fee shall be charged to foreign members. Each registered member shall receive a card of membership and be furnished a set of the transactions.

### ABSTRACTS, PAPERS AND DISCUSSIONS.

3. Contributors are required to forward abstracts of their papers, not to exceed 600 words each, to be in the hands of the Secretary-General not later than the 10th of July, 1893. These abstracts shall be translated into English, French, Spanish, and Portuguese, and shall be published in advance of the meeting for the convenience of the Congress, and no paper shall be placed upon the programme which has not been thus presented by abstract. Papers and discussions will be printed in the language in which they may be presented. All papers read in the Sections shall be surrendered to the Secretaries of the Sections ; all addresses read in the General Session shall be surrendered to the Secretary-General as soon as read ; and all discussions shall be at once reduced to writing by the participants.

### INCORPORATION.

4. The Chairman of the Committee on Organization shall cause the Congress to be incorporated under the laws of Ohio, and fifteen trustees shall be elected in accordance therewith, who by by-laws and through the Executive Committee shall supervise all receipts and disbursements by the Treasurer in accordance with the laws of Ohio. The President, Secretary-General, Treasurer, the member of the International Executive Committee for the United States, and Chairmen of Sections, shall be *ex-officio* members of the Board of Trustees.

### FOREIGN NOMINATIONS.

5. All nominations by the International Executive Committee must be in the hands of the Chairman of the Committee on Organization by June 1, 1892, and in default thereof the Committee on Organization shall elect officers for countries thus delinquent.

THE ORGANIZATION OF SECTIONS.

6. The officers of each section shall consist of —— Honorary Chairmen, who shall be residents of the constituent countries of the Congress; one Executive Chairman, who shall organize the work of the Section, direct its deliberations, and deliver an inaugural address at its opening session; one English-speaking Secretary and one Spanish-speaking Secretary, residents of the United States, who shall coöperate with the Executive Chairman in conducting the correspondence of the Section; and there shall be one secretary for each section, resident in each additional constituent country of the Congress.

DOMESTIC AUXILIARY COMMITTEE.

7. The Auxiliary Committee for the United States shall be elected by the Committee on Organization, and shall consist of one member for each local medical society, or, in the absence of medical organization, then one in each considerable center of population, which Auxiliary Committee shall coöperate with the Committee on Organization and with the General Officers in promoting the welfare of the Congress. Nominations for the Auxiliary Committee shall be made by members of the Committee on Organization, each for his own State, except that in the failure of any member to make such nomination by January 1, 1893, or in the inadequacy of the same, the Chairman of the Committee on Organization shall supply the deficiency.

EXECUTIVE COMMITTEE.

8. The Board of Trustees shall designate seven members, including the President, Treasurer, Secretary-General, and member of the International Executive Committee for the United States, who shall comprise an Executive Committee which shall transact all business of the Congress *ad interim* in accordance with By-laws adopted by the Board of Trustees.

AMENDMENTS.

9. Amendments to these Regulations can be made only by the International Executive Committee on a majority vote, ten members constituting a quorum, at any meeting of the Congress.

Pursuant to the Laws of Ohio and the Regulations adopted as above, and in accordance with nominations by the Committee on Permanent Organization, the Incorporators elected fifteen Trustees, as follows:

Dr. W. T. Briggs, Tennessee; Dr. Geo. F. Shrady, New York; Dr. P. O. Hooper, Arkansas; Dr. S. S. Adams, D. C., Dr. H. O. Marcy, Massachusetts; Dr. J. F. Kennedy, Iowa; Dr. H. T. Holton, Vermont; Dr. L. S. McMurtry, Kentucky; Dr. N. S. Davis,

Illinois; Dr. Levi Cooper Lane, California; Dr. I. N. Love, Missouri; Dr. Hunter McGuire, Virginia; Dr. J. C. Culbertson, Illinois; Dr. A. Walter Suiter, New York; Dr. C. H. Mastin, Alabama.

Drs. L. S. McMurtry (Ky.), I. N. Love (Mo.), and W. W. Potter (N. Y.) were designated to act as members of the Executive Committee.

The organization of the Congress is complete in British North America, the British West Indies, the Spanish West Indies, Guatemala, Nicaragua, United States of Colombia, Brazil, Uruguay, Venezuela and the Argentine. It is confidently expected that the nominations from the remaining countries will be in by June.

It is expected to announce the completed organization of the Congress, its sections and auxiliary committees, domestic and foreign, by July 1, 1892.

On behalf of the Committee on Permanent Organization,

CHARLES A. L. REED, *Chairman.*

J. W. CARHART, *Secretary.*

CINCINNATI, March 17, 1892.

---

SOUTHERN PINES, Moore county, N. C., is located on the highest sand ridge in the South. It has the driest climate, and the greatest amount of ozone is guaranteed by the forests of long-leaf pine which covers a territory of fifty miles in each direction.

Last year a delegation of Northern physicians was invited to examine and report on the place as to its adaptability for those suffering with throat and lung troubles. The delegation reported, through Dr. W. C. Wile, of Danbury, Conn., editor of the *New England Medical Journal.* At the end of the report the doctor says: "In conclusion, I am satisfied that Southern Pines possesses more of the qualifications of a genuine health resort, especially for those who are afflicted with pulmonary diseases and all of those of the air-passages, than any other place with which I am acquainted or have read about." With such information, we advise our readers who desire to send consumptive patients South, to make further investigation, for we have every reason to believe that Southern Pines must be a very desirable Winter resort. Hon. J. T. Patrick, Secretary of the Southern Bureau of Information, can give reliable information. His office is at Raleigh, N. C., which is near Southern Pines. It is Mr. Patrick's special business to furnish information of the South to those seeking it. What he says can be relied on

Messrs. P. Blakiston & Co., publishers, 1012 Walnut street, Philadelphia, announce that they have just ready from the press the following works, viz.: A Treatise on Diseases of the Lungs and Pleuræ, by the late Dr. Wilson Fox, F. R. S. P., Physician-in-Ordinary to Her Majesty the Queen. This book will consist of 1,200 octavo pages, with illustrations, and will be furnished in cloth at the net price of $8.00, prepaid to any address. Subscriptions should be sent direct to the publishers.

Also the following: Diseases of the Throat, Nose and Ear, with thirty-eight handsome colored illustrations printed in the text. A clinical manual for students and practitioners, by P. McBride, M. D., F. R. C. P., Edin., Fellow of the Royal Society, Surgeon to Ear and Throat Department of Royal Infirmary, Edinburgh, etc. In this work it has been the author's aim, while omitting nothing of importance, to so apportion the space as to attain the greatest usefulness to the general practitioner. To this end common diseases have been discussed at greater length, and the purpose preserved throughout of bringing the main and modern facts within a volume of reasonable dimensions. Special attention is requested to the method and quality of illustration by colored lithographs. These are all original, and most of them were drawn by Mr. J. Bayne from patients of the author. The fine execution of these drawings, under the direct superintendence of Drs. Semon and G. H. Mackenzie, is in full keeping with the new and comprehensive character of the book.

---

The Pope Manufacturing Co., 221 Columbus avenue, Boston, Mass., offers 100 prizes on the subject of good roads. These 100 prizes consist of an equal number of Columbia Bicycles to be distributed among the authors of the best published essays on the subject which we named. Each essay must not be less than 500 words in length, and it must be published in some paper or magazine between now and the first of May. All competing essays must be sent to the above address. Further instructions will be given upon application to the Pope Manufacturing Co.

